# Peri-Procedural Safety of GLP-1 Receptor Agonists in Elective Endoscopy: A Multicenter Retrospective Cohort Study

**DOI:** 10.3390/jcm14176147

**Published:** 2025-08-30

**Authors:** Harsimran Kalsi, Raghav Bassi, Hussein Noureldine, Kobina Essilfie-Quaye, Carson Creamer, Mohammad Abuassi, Robyn Meadows, Tony S. Brar, Yaseen Perbtani

**Affiliations:** 1 Department of Internal Medicine, University of Central Florida College of Medicine, Orlando, FL 32827, USA; 2 Department of Internal Medicine, HCA Florida North Florida Hospital, Gainesville, FL 32605, USA; 3 Department of Internal Medicine, The University of Oklahoma Health Sciences Center, Oklahoma City, OK 73104, USA; 4 GME Research Statistical Department, HCA Healthcare, Brentwood, TN 37027, USA; 5 Center for Advanced Therapeutic Endoscopy (CATE), HCA Florida North Florida Hospital, Gainesville, FL 32605, USA; 6 Digestive Disease Associates, HCA Florida North Florida Hospital, Gainesville, FL 32605, USA

**Keywords:** GLP-1 agonists, endoscopy, colonoscopy, anesthesia, aspiration

## Abstract

**Background and Aims**: Glucagon-like peptide-1 receptor agonists (GLP-1 RAs) delay gastric emptying, raising concerns about periprocedural safety in elective endoscopy. We aimed to evaluate the association between pre-procedural GLP-1 RA use and post-procedural complications such as aspiration pneumonia. **Methods**: In this retrospective cohort study, adults (18–89 years) undergoing outpatient esophagogastroduodenoscopy or colonoscopy within the HCA Healthcare network from 1 July 2021 to 31 March 2024 were identified. Patients were classified as GLP-1 RA users (*n* = 953) or non-users (*n* = 3289) based on home medication records. Primary outcomes included aspiration, post-procedural oxygen requirement, hypotension, hospitalization, ICU admission, length of stay, and all-cause inpatient mortality. Multivariable logistic and negative-binomial regression models, incorporating an interaction term for anesthesia type, were adjusted for age, sex, body mass index, ASA class, and key comorbidities. **Results**: No aspiration events were reported in either group. GLP-1 RA use was associated with lower odds of post-procedural oxygen requirement (OR 0.43, 95% CI 0.25–0.76), hospitalization (OR 0.73, 95% CI 0.39–1.36), and mortality (0.1 vs. 0.9%, *p* = 0.014), and a shorter hospital stay (IRR 0.54, 95% CI 0.40–0.71). Rates of hypotension and ICU admission were similar between both groups. In anesthesia-stratified analysis among GLP-1 RA users, those receiving MAC/MS had higher odds of hospitalization compared with GA (OR 1.87, 95% CI 1.23–2.85, *p* = 0.003), whereas other outcomes were not significant. **Conclusions**: Pre-procedural GLP-1 RA therapy was not associated with increased peri-procedural complications. Although hospitalization was more frequent with MAC/MS, this difference did not extend to other clinically significant outcomes. Further prospective studies are needed to clarify the clinical implications of anesthesia choice.

## 1. Introduction

Glucagon-like peptide-1 receptor agonists (GLP-1 RAs) are established treatments for type 2 diabetes mellitus and obesity [1]. This class of injectable and recently approved oral medications improve glycemic control and facilitate weight loss through enhanced insulin secretion and glucagon suppression [1,2]. A recognized pharmacologic effect of GLP-1 RAs is delayed gastric emptying, mediated via central and peripheral cholinergic pathways [3,4]. This may increase the risk of retained gastric contents and aspiration under procedural sedation [4,5].

Professional societies provide varying guidelines on the peri-endoscopic management of GLP-1 RAs. The American Gastroenterological Association (AGA) does not mandate routine discontinuation of GLP-1 RAs before elective endoscopy [6]. Whereas the American Society of Anesthesiologists (ASA) and American Society for Gastrointestinal Endoscopy (ASGE) recommend withholding therapy due to potential aspiration risks [7,8]. Specifically, both ASA and ASGE advise a 24 h hold for daily formulations or 7-day hold for weekly formulations, although these guidelines primarily rely on theoretical risks and limited empirical evidence [7,8]. Furthermore, uncertainty regarding aspiration risk among GLP-1 RA users may lead anesthesia providers to preferentially select general anesthesia (GA) over monitored anesthesia care (MAC), potentially resulting in unnecessary intubation and associated risks [9]. Clear evidence supporting this cautious approach remains limited, emphasizing the importance of evaluating anesthesia subtype as a potential factor influencing clinical decision-making and patient outcomes.

Given the rapid adoption of GLP-1 RAs and the potential clinical and logistical consequences of drug withholding, including glycemic destabilization, procedure delays, and increased healthcare utilization, there remains a critical need for robust clinical evidence [10]. We conducted a multicenter retrospective cohort study within the HCA Healthcare network to evaluate the association between pre-procedural GLP-1 RA use and peri-procedural complications, and to determine if anesthesia subtype impacts this relationship.

## 2. Methods

We conducted a retrospective cohort study utilizing the national HCA Healthcare database to assess the impact of GLP-1 RA use on post-endoscopic outcomes. Patient data were extracted from electronic medical records across multiple healthcare centers under HCA across the United States of America.

### 2.1. Study Population

We included patients aged 18 to 89 years with type 2 diabetes mellitus or obesity who underwent elective esophagogastroduodenoscopy (EGD) and/or colonoscopy between 1 July 2021, and 31 March 2024. Elective procedures were defined as non-emergent, pre-scheduled outpatient procedures performed for diagnostic or therapeutic purposes. While these procedures were elective in nature, they were conducted in inpatient setting due to the patients’ higher baseline comorbidities and overall clinical risk profile. All procedures were scheduled in advance and not performed on an urgent or emergent basis (Figure S1).

Patients were categorized into two groups based on home use of GLP-1 RAs at the time of the procedure. The GLP-1 RA group comprised individuals with an active prescription of GLP-1 RA (e.g., semaglutide, liraglutide, dulaglutide, exenatide, albiglutide, lixisenatide) on their home medication list, while the non-GLP-1 RA group included patients with no reported use or active prescription of these medications. Patients were excluded if they underwent urgent or emergent procedures, had incomplete medical records, or were under 18 years of age. Initially, 8778 records were identified for screening, and 4242 patients met the inclusion criteria. Procedural distribution comprised 39.12% colonoscopies and 60.88% EGDs.

### 2.2. Data Collection

Baseline demographic and clinical characteristics were collected, including age, sex, race, body mass index (BMI), smoking status, alcohol use, and comorbidities such as diabetes mellitus (DM), heart failure, chronic kidney disease (CKD), obstructive sleep apnea (OSA), chronic obstructive pulmonary disease (COPD), coronary artery bypass surgery (CABG), coronary artery disease (CAD), gastroparesis, opioid use, and stroke. Anesthesia type was also recorded and categorized as GA, MAC, or moderate sedation (MS). For analytical purposes, MAC and MS were grouped together given the similarity in sedation depth and the frequent overlap in documentation and clinical practice.

The data used in our analysis was extracted using specialized software built in the electronic medical records (Meditech Magic and Meditech Expanse) to ensure efficiency and accuracy. During this process, all patient information was de-identified, maintaining strict confidentiality and compliance with privacy standards. None of the investigators had access to patient-protected health information.

### 2.3. Outcomes

Primary outcomes included aspiration, post-procedural oxygen requirement, post-procedural hypotension, intensive care unit (ICU) admission, ICU length of stay (LOS), and mortality. All adverse events occurred during index hospital admission following the elective procedure and were temporally related to the procedure. Aspiration was defined as the inhalation of gastric contents into the respiratory tract, confirmed by clinical documentation, ICD codes, or post-procedural radiographic evidence of aspiration pneumonitis or pneumonia. Post-procedural oxygen requirement was defined as the need for intubation or supplemental oxygen via BiPAP, CPAP, or nasal cannula following the procedure. Hypotension was defined as documented systolic blood pressure of <80 mmHg occurring within 24 h after the procedure. Hospitalization referred to any unplanned admission to the hospital post-procedure, including direct admission from the endoscopy unit, resulting in inpatient care during the same encounter. ICU admission was defined as any unplanned transfer to the ICU after the procedure during the same clinical encounter, and ICU LOS was reported in days. Mortality was defined as all cause in-hospital death or discharge to hospice during the index hospitalization. The underlying causes of hospitalization, ICU admission and mortality were not evaluated in this study, as this fell outside the predefined scope of our study design.

To assess whether the association between GLP-1 RA use and post endoscopic outcomes varied by anesthesia type, we performed a subgroup analysis stratified by GA versus MAC/MS on post-procedural outcomes within the GLP-1 RA users. Key outcomes included aspiration, post-procedural oxygen requirement, post-procedural hypotension, ICU admission, and ICU LOS.

### 2.4. Statistical Analysis

Descriptive statistics were used to compare baseline characteristics between GLP-1 RA users and non-users. Continuous variables were reported as means with standard deviations (SDs) or medians with interquartile ranges (IQRs) and analyzed using independent t-tests or Mann–Whitney U tests, as appropriate. Categorical variables were reported as counts with percentages and compared using chi-square or Fisher’s exact tests as warranted by the data distribution. To maintain analytical rigor and avoid overinterpretation of spurious results, logistic regression analyses were only pursued if these initial tests indicated statistically significant results (*p* < 0.05).

For outcomes showing significant associations, logistic regression was used for binary outcomes such as post-procedural oxygen requirement and ICU admission, with results expressed as odds ratios (ORs) and 95% confidence intervals (CIs). Firth correction was applied for rare events. Fisher’s exact test was used for categorical outcomes with small cell sizes (e.g., post-procedural hypotension). Negative binomial regression was used to model ICU LOS, reported as incident rate ratios (IRRs).

A stratified analysis was also performed within the GLP-1 RA group to assess whether anesthesia type (GA vs. MAC/MS) influenced post-procedural outcomes. All statistical analyses were conducted using SAS (version 9.4M7) and R (version 4.4.1) software, with a two-sided *p*-value of < 0.05 considered statistically significant.

### 2.5. Ethical Considerations

All research was performed according to the Declaration of Helsinki. This study was conducted in accordance with the ethical guidelines of the institution and received approval from the HCA Healthcare Institutional Review Board (Protocol Code 24-1899; Approval Date: 29 May 2024). Due to the retrospective nature of this study, patient consent was waived. All patient data were anonymized to ensure confidentiality and compliance with ethical research standards.

## 3. Results

### 3.1. Demographic and Clinical Characteristics

A total of 4242 patients were included, comprising 953 (22.5%) GLP-1 RA users and 3289 (77.5%) non-users. Mean age (61.83 ± 11.56 vs. 61.16 ± 14.58 years, *p* = 0.142) and proportion of female patients (55.8% vs. 54.6%, *p* = 0.517) were similar between groups. Significant differences in race distribution were observed among users and non-users (*p* = 0.0004). GLP-1 RA users included a higher proportion of African Americans (15.6% vs. 13.1%), and fewer Hispanic individuals (7.1% vs. 11.6%).

GLP-1 RA users had a significantly higher BMI compared to non-users (36.81 ± 9.73 vs. 30.65 ± 8.98, *p* < 0.0001). Smoking (10.8 vs. 17.0%, *p* < 0.0001) and alcohol consumption (0.6 vs. 2.7%, *p* = 0.0002) were significantly less common in GLP-1 RA users.

Charlson Comorbidity Index scores were higher among GLP-1 RA users (median 2.0, [IQR 1.0–3.0] vs. median 1.0 [IQR 0.0–2.0]; *p* < 0.0001). Several comorbidities, including diabetes (88.4 vs. 26.4%), coronary artery disease (21.8 vs. 16.5%), chronic kidney disease (17.2 vs. 10.7%), gastroparesis (4.8 vs. 2.2%), heart failure (11.4 vs. 8.7%), and obstructive sleep apnea (25.2 vs. 10.1%) were significantly more prevalent among GLP-1 RA users (all *p* < 0.01). No significant differences were found in COPD, opioid use, or stroke history. Additional demographic, clinical, and anesthesia-related details are presented in Table 1.

### 3.2. Anesthesia Characteristics

**Figure 1:**
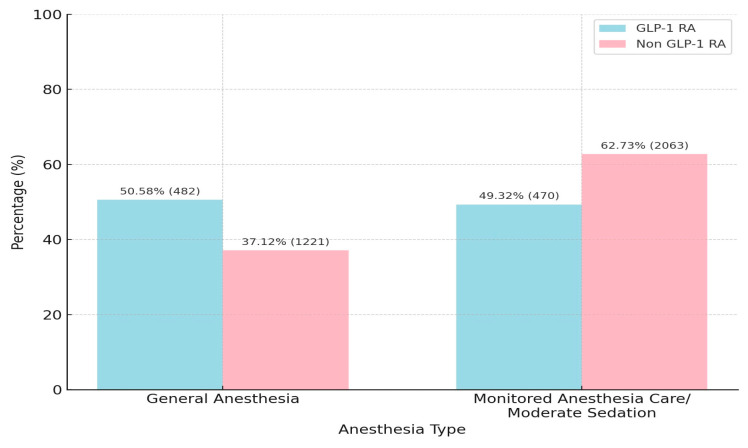
Distribution of anesthesia types among GLP-1 RA users and non-users. GLP-1 RA users were more likely to receive general anesthesia, whereas non-users more frequently underwent monitored anesthesia care/moderate sedation. Percentages indicate the distribution within each group (*p* < 0.0001).

GA was administered significantly more frequently among GLP-1 RA users (50.6 vs. 37.1%, *p* < 0.0001). Conversely, non-users received MAC/MS more commonly (62.7 vs. 49.3%) (Figure 1).

### 3.3. Clinical Outcomes

**Figure 2:**
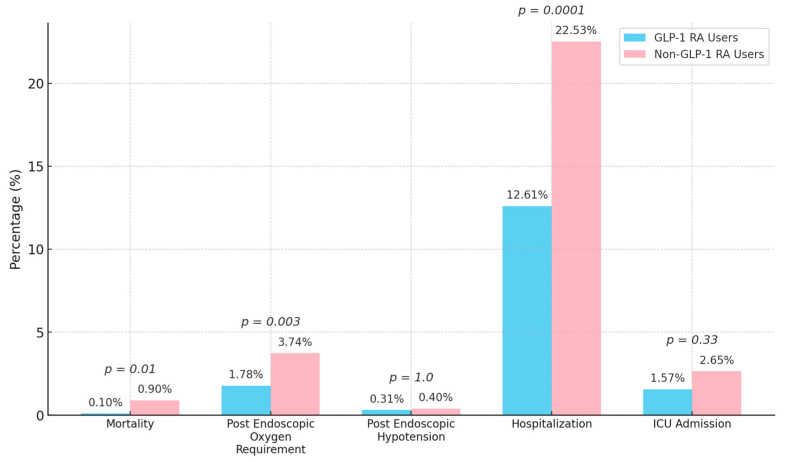
Comparison of post-endoscopic clinical outcomes between GLP-1 RA users and non-users. GLP-1 RA users demonstrated significantly lower rates of mortality, post-procedural oxygen requirement, and hospitalization compared to non-users.

No aspiration events occurred in either group. Mortality was significantly lower among GLP-1 RA users (0.1 vs. 0.9%, *p* = 0.01) (Figure 2).

**Figure 3:**
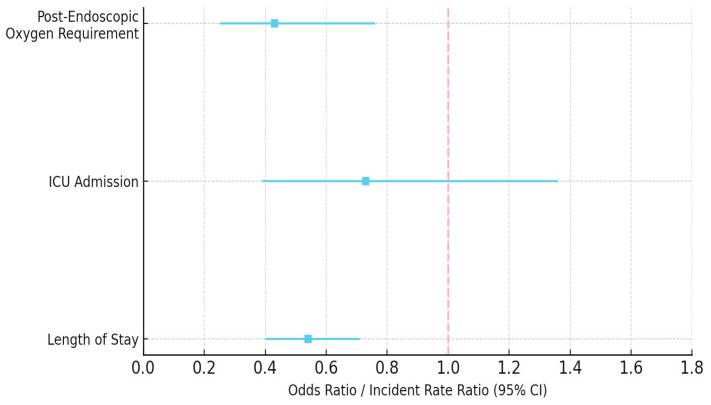
Forest plot of odds ratio and incident rate ratios for post-endoscopic outcomes in GLP-1 RA users vs. non-users. Includes post-procedural oxygen requirement, ICU admission, and length of stay. The dashed line represents the null value (OR/IRR = 1.0).

The incidence of post-procedural hypotension was similarly low between GLP-1 RA users and non-users (0.31 vs. 0.40%, *p* = 1.0). Post-procedural oxygen requirements (1.8 vs. 3.7%, OR 0.43, 95% CI: 0.25–0.76, *p* = 0.003) and hospitalization (12.6 vs. 22.5%, *p* < 0.0001) were significantly lower among GLP-1 RA users. ICU admission rates were similar between groups (1.6 vs. 2.7%, OR 0.73, 95% CI: 0.39–1.36, *p* = 0.33). Length of stay was significantly shorter for GLP-1 RA users (mean 0.44 ± 1.76 days vs. 0.96 ± 2.98 days, IRR 0.54, 95% CI: 0.40–0.71, *p* < 0.0001) (Table 2, Figure 2 and Figure 3).

### 3.4. Effect of Anesthesia on Clinical Outcomes

Among GLP-1 RA users, no aspiration events occurred with either anesthesia type. There were no significant differences in post-procedural oxygen requirement or hypotension between GA and MAC/MS groups. However, hospitalization rates were significantly more frequent in GLP-1 RA users who received MAC/MS compared to GA (OR 1.87, 95% CI: 1.23–2.85, *p* = 0.003). No significant differences in ICU admission rates or length of stay by anesthesia type were observed (ICU admission, *p* = 0.46; length of stay IRR 1.67, 95% CI: 1.03–2.72, *p* = 0.39) (Table 3).

## 4. Discussion

This multicenter observational cohort study assessed peri-procedural safety outcomes among patients using GLP-1 RAs undergoing elective endoscopy. Our analysis demonstrated no increased risk of peri-procedural complications in GLP-1 RA users compared to non-users. Notably, GLP-1 RA users experienced significantly lower rates of mortality, post-procedural oxygen requirements, hospitalization, and shorter hospital stays.

While our findings demonstrate favorable safety outcomes, existing literature on peri-procedural aspiration risks associated with GLP-1 receptor agonists remains conflicting. Yeo et al. reported a modest but statistically significant increase in aspiration pneumonia among GLP-1 RA users undergoing gastrointestinal endoscopy (0.83% incidence vs. 0.63% in non-users; HR 1.33, 95% CI 1.02–1.74) [11]. However, this result contrasts with other studies [12,13]. For instance, Velji-Ibrahim et al. using the same electronic database, found no significant differences in post-endoscopy aspiration pneumonitis or pneumonia rates between GLP-1 RA users and non-users (HR 0.92 and 1.01, respectively) [13]. Similarly, large-scale studies by Alkabbani et al. have observed no increased aspiration risk among GLP-1 RA users compared to other antidiabetic agents [5]. Collectively, the weight of existing evidence including real-world cohorts and meta-analyses does not consistently support an elevated aspiration risk attributable to GLP-1 RAs, suggesting aspiration concerns may be overstated.

Current guideline recommendations for withholding GLP-1 RAs before elective endoscopy are primarily based on theoretical risks and limited empirical evidence. The ASA and the ASGE advocate withholding GLP-1 RAs pre-procedure due to concerns over delayed gastric emptying [7,8]. However, these guidelines rely heavily on case reports and small observational studies documenting residual gastric content (RGC), rather than robust prospective trials demonstrating actual aspiration events [11,14]. Both societies acknowledge the lack of direct evidence linking GLP-1 RAs to increased aspiration risk, highlighting the precautionary nature of these guidelines.

Several large observational studies and systematic reviews further illustrate that while GLP-1 RAs may delay gastric emptying, this does not translate into significantly higher aspiration rates [15]. Singh et al. conducted a comprehensive meta-analysis finding no association between pre-procedural GLP-1 RA use and pulmonary aspiration (OR 21.06, 95% CI 0.13–3379.01, *p* = 0.24 [16]. More recently, Tarar et al. performed a large-scale meta-analysis of over 100,000 patients, similarly reporting no significant increase in aspiration risk among GLP-1 RA users (OR 1.26, 95% CI 0.86–1.87), despite higher odds of RGC and increased rates of aborted or repeated procedures [17]. Although GLP-1 RA users more frequently exhibited increased RGC, reduced mucosal visibility, and higher rates of prematurely terminated endoscopic procedures, these factors did not translate into a higher risk of aspiration [15,16,17,18]. Consequently, universal withholding of GLP-1 RAs before elective endoscopy may be unnecessary. Pending robust prospective data, current guidelines based on theoretical concerns should be interpreted cautiously, highlighting the importance of carefully weighing patient-specific risks rather than routinely withholding GLP-1 RAs.

Although our study found no increased peri-procedural complications with GLP-1 RA use, the anesthesia choice remains an important clinical consideration guided by individual patient risk factors. Conservative guidelines on delayed gastric emptying have led to increased utilization of GA among GLP-1 RA users [19]. However, our subgroup analysis revealed no additional periprocedural safety benefits from routine GA compared to MAC/MS, indicating routine GA might represent an unnecessary precaution without clear evidence of improved outcomes. Therefore, individualized risk assessments that account for patient-specific factors such as their clinical presentation, sedation tolerance, respiratory comorbidities, and procedural complexity may provide a balanced, resource-conscious anesthesia management strategy.

In this context, our subgroup analysis did identify higher hospitalization rates among GLP-1 RA users undergoing procedures with MAC/MS compared to general anesthesia. While this finding is intriguing, it is likely influenced by residual confounding, including variations in provider judgment or procedural complexity. All patients were referred from ambulatory clinics to hospital settings for elective endoscopy due to their high-risk profiles, and GA may have been preferentially selected by anesthesiologists to mitigate perceived procedural risks. Factors such as incomplete sedation, patient anxiety, or logistical delays could have contributed to hospitalization in the MAC/MS group, despite these patients being perceived as lower risk. Importantly, other key peri-procedural outcomes, including aspiration, hypoxia, hypotension, and ICU admission, did not differ by anesthesia type.

Moreover, over 90% of cases occurred prior to release of the 2023 ASA guidelines recommending GLP-1 RA withholding, suggesting that GA use was not primarily driven by concerns for GLP-1 RAs related aspiration. Thus, while GA was more commonly used, its association with lower hospitalization likely reflects selection bias rather than protective effect. Our results suggest careful anesthesia consideration without universally favoring GA. Prospective studies addressing these confounders will further clarify optimal sedation strategies.

Our findings should be interpreted within the context of several strengths and limitations. Strengths include the large multicenter cohort enhancing generalizability and the outcomes assessed are clinically relevant. Additionally, our study explicitly compares GA and MAC/MS outcomes in GLP-1 RA users, addressing a notable clinical gap and supporting an individualized anesthesia management approach.

However, several limitations warrant consideration. The retrospective design and reliance on electronic records introduce potential confounding and misclassification biases. Specifically, we could not verify whether GLP-1 RAs were withheld before endoscopy, limiting definitive conclusions regarding the absence of aspiration events. However, because the majority of data collection occurred before the publication of the 2023 ASA guidelines recommending GLP-1 RA withholding, it is reasonable to presume that most of the patients continued their medication [7]. This strengthens the real-world applicability of our findings.

Additionally, we analyzed patients undergoing EGD and colonoscopy collectively rather than separately examining these procedures. While increased RGC has been documented predominantly during EGD [5,20], recent large-scale studies and systematic reviews have demonstrated comparable peri-procedural respiratory safety profiles between EGD and colonoscopy among GLP-1 RA users, providing justification for our combined analytical approach [13,16]. Nevertheless, procedure-specific risks and benefits may still exist, warranting careful interpretation of these results. While Beran et al. reported inadequate bowel preparation in GLP-1 RA users, we did not directly assess RGC or bowel preparation adequacy, which could influence procedural quality despite not correlating with increased aspiration [21].

Importantly, the finding of a lower complication rate among patients in the GLP-1 RA group, despite their higher burden of comorbidities, may be attributable to residual confounding from unmeasured variables. Although the multivariable analyses adjusted for baseline clinical characteristics, it is possible that provider-level factors such as heightened procedural vigilance, the preferential selection of general anesthesia, and enhanced pre-procedural optimization for patients perceived to be at higher risk. Such clinical behaviors were not captured within the dataset but may have played a role in mitigating peri-procedural complications. Similarly, the observed mortality in both groups is likely attributable to baseline comorbidities, as our definition encompassed all-cause mortality during the index admission rather than procedure-specific mortality.

Lastly, the increased hospitalization rate observed among GLP-1 RA users receiving MAC/MS compared to general anesthesia may reflect residual confounding inherent to the retrospective design, including unmeasured patient-specific risks or provider biases influencing anesthesia selection. Prospective studies are needed to confirm these findings.

In conclusion, this large multicenter observational study found no evidence of increased peri-procedural complications in GLP-1 RA users undergoing elective endoscopy, including aspiration, oxygen requirements, hospitalization rates, and mortality. Furthermore, routine GA offered no clear safety advantage over MAC/MS, suggesting current generalized anesthesia guidelines may not be warranted. Our results advocate for an individualized, patient-centered approach to peri-endoscopic management of GLP-1 RAs, emphasizing clinical judgment and personalized risk assessment.

## Figures and Tables

**Table 1:** Baseline demographics, clinical characteristics, and procedural details of GLP-1 RA users vs. non-users.

	Use of GLP-1 Receptor Agonist		*p* Value
	Yes (*n*) = 953	No (*n*) = 3289	
Demographic Characteristics			
Age, mean (SD)	61.83 (11.56)	61.16 (14.58)	0.142 ^a^
Female, % (*n*)	55.82 (532)	54.64 (1797)	0.517 ^b,x^
Male, % (*n*)	44.18 (421)	45.36 (1492)	
Race, % (*n*)			
African American	15.63 (149)	13.10 (431)	0.0004 ^b,x^
Hispanic	7.14 (68)	11.55 (380)	
White	70.93 (676)	68.41 (2250)	
Others	6.30 (60)	6.93 (228)	
Lifestyle Factors			
Body mass index, mean (SD)	36.81 (9.73)	30.65 (8.98)	<0.0001 ^a^
Smoking status, % (*n*)			
Never	57.19 (545)	54.42 (1790)	<0.0001 ^b,x^
Former	32.00 (305)	28.58 (940)	
Current	10.81 (103)	17.00 (559)	
Alcohol use, % (*n*)	0.63 (6)	2.65 (87)	0.0002 ^c^
Comorbidities, % (*n*)			
Coronary artery disease	21.83 (208)	16.51 (543)	0.0002 ^b^
Coronary artery bypass surgery	19.62 (187)	14.90 (490)	0.0005 ^b^
Chronic kidney disease	17.21 (164)	10.73 (353)	<0.0001 ^b^
Chronic obstructive pulmonary disease	7.45 (71)	8.18 (269)	0.466 ^b^
Diabetes mellitus	88.35 (842)	26.36 (867)	<0.0001 ^b^
Gastroparesis	4.83 (46)	2.22 (73)	<0.0001 ^b^
Heart failure	11.44 (109)	8.67 (285)	0.009 ^b^
Obstructive sleep apnea	25.18 (240)	10.06 (331)	<0.0001 ^b^
Opioid use	0.31 (3)	0.40 (13)	1.000 ^c^
Stroke, chronic	0.31 (3)	0.15 (5)	0.390 ^c^
Charlson comorbidity index, median (IQR)	2.00 (1.00, 3.00)	1.00 (0.00, 2.00)	<0.0001 ^d^
Anesthesia Type, % (*n*)			
General anesthesia	50.58 (482)	37.12 (1221)	<0.0001 ^b,x^
Monitored anesthesia care/Moderate sedation ^y^	49.32 (470)	62.73 (2063)	

^a^ Independent samples t-test; ^b^ Chi square test; ^c^ Fisher’s exact test; ^d^ Mann–Whitney U-test; ^x^ For categorical variables with multiple categories (gender, race, smoking status, and anesthesia type), the *p*-value reflects the overall distribution across all categories, not individual group comparisons; ^y^ Monitored anesthesia care and moderate sedation were combined for statistical analysis due to their overlapping clinical applications and similar procedural contexts.

**Table 2:** Clinical outcomes in GLP-1 RA users vs. non-GLP-1 RA users.

Clinical Outcome	GLP-1 RA Users N (%) or Mean (SD)	Non GLP-1 RA Users N (%) or Mean (SD)	Odds Ratio (95% Confidence Interval, *p* Value)
Aspiration	0	0	–
Mortality	1 (0.1%)	28 (0.9%)	(*p* = 0.01) ^a^
Post Endoscopic Oxygen Requirement	17 (1.78%)	123 (3.74%)	0.43 (0.25–0.76, 0.003)
Post Endoscopic Hypotension	3 (0.31%)	13 (0.40%)	(*p* = 1.0) ^b^
Hospitalization	120 (12.61%)	740 (22.53%)	(*p* = 0.0001) ^a^
ICU Admission	15 (1.57%)	87 (2.65%)	0.73 (0.39–1.36, 0.33)
Length of Stay	0.44 ± 1.76	0.96 ± 2.98	0.54 (0.40–0.71, <0.0001) ^c^

^a^ Chi-square test; ^b^ Fisher exact test; ^c^ incident rate ratio; GLP-1 RA, glucagon-like peptide-1 receptor agonist; SD, standard deviation.

**Table 3:** Comparison of clinical outcomes in GLP-1 RA users by anesthesia type.

Clinical Outcomes	GLP-1 RA Users (N = 952)		Odds Ratio (95% Confidence Interval, *p* Value)
	Monitored Anesthesia Care/Moderate Sedation N (%) or Mean (SD)	General Anesthesia N (%) or Mean (SD)	
Post Endoscopic Oxygen Requirement	9 (1.91%)	8 (1.66%)	(*p* = 0.76) ^a^
Post Endoscopic Hypotension	1 (0.21%)	2 (0.41%)	(*p* = 1.00) ^a^
Hospitalization	74 (15.74%)	46 (9.54%)	1.87 (95% CI: 1.23–2.85, 0.003)
ICU Admission	6 (1.28%)	9 (1.87%)	(*p* = 0.46) ^a^
Length of Stay	0.56 ± 2.08	0.33 ± 1.38	1.67 (95% CI: 1.03–2.72, 0.39) ^b^

^a^ Fisher exact test; ^b^ incident rate ratio; ICU, intensive care unit; GLP-1 RA, glucagon-like peptide-1 receptor agonist; SD, standard deviation.

## Data Availability

The data that support the findings of this study are available from HCA Healthcare; however, restrictions apply to the availability of the data, which were used under institutional license for the current study, and so are not publicly available. Data may be available from the authors upon reasonable request and with the permission of HCA Healthcare.

## Supplementary Materials

The following supporting information can be downloaded at: https://www.mdpi.com/article/10.3390/jcm14176147/s1, Figure S1: Enrollment flowchart to illustrate the inclusion and exclusion process for the study cohort.

## Abbreviations

AGA	American Gastroenterological Association
ASA	American Society of Anesthesiologists
ASGE	American Society for Gastrointestinal Endoscopy
BMI	Body Mass Index
BiPAP	Bilevel Positive Airway Pressure
CABG	Coronary Artery Bypass Grafting
CAD	Coronary Artery Disease
CI	Confidence Interval
CKD	Chronic Kidney Disease
COPD	Chronic Obstructive Pulmonary Disease
CPAP	Continuous Positive Airway Pressure
DM	Diabetes Mellitus
EGD	Esophagogastroduodenoscopy
GA	General Anesthesia
GLP-1 RA	Glucagon-Like Peptide-1 Receptor Agonist
ICU	Intensive Care Unit
IRB	Institutional Review Board
IRR	Incidence Rate Ratio
IQR	Interquartile Range
LOS	Length of Stay
MAC	Monitored Anesthesia Care
MS	Moderate Sedation
OR	Odds Ratio
OSA	Obstructive Sleep Apnea
RGC	Residual Gastric Content
SD	Standard Deviation
