# Supplementary figures and images for: Peri-Procedural Safety of GLP-1 Receptor Agonists in Elective Endoscopy: A Multicenter Retrospective Cohort Study
Source: J Clin Med. 2025 Aug 30;14(17):6147. doi: 10.3390/jcm14176147 (PMC12429542; doi:10.3390/jcm14176147)

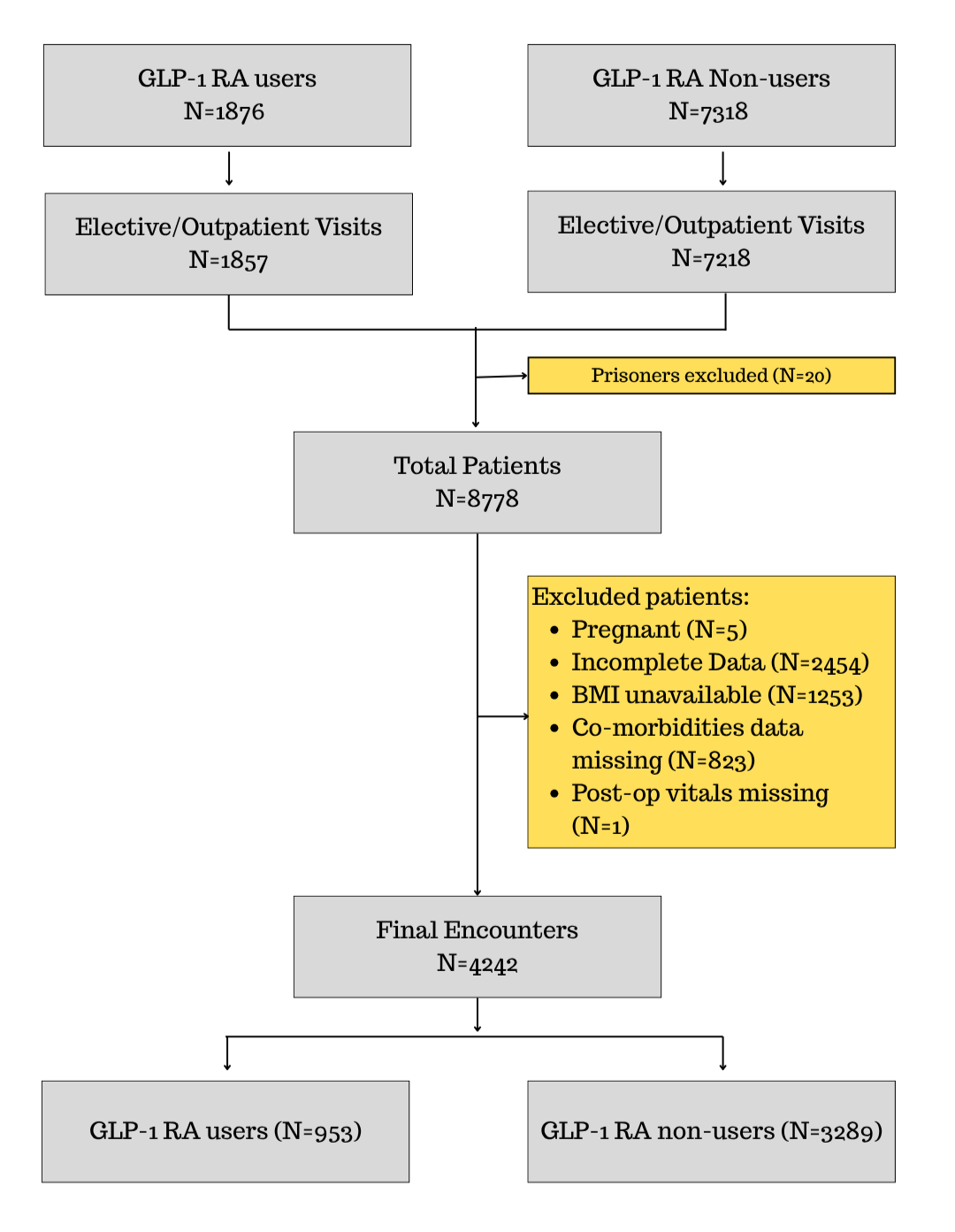

Supplement: Supplementary file 1 [file jcm-14-06147-s001.zip › jcm-3772464-supplementary.png]
